# MiR-590-3p promotes proliferation and metastasis of colorectal cancer via Hippo pathway

**DOI:** 10.18632/oncotarget.19487

**Published:** 2017-07-22

**Authors:** Zhen-Qiang Sun, Ke Shi, Quan-Bo Zhou, Xiang-Yue Zeng, Jinbo Liu, Shuai-Xi Yang, Qi-San Wang, Zhen Li, Gui-Xian Wang, Jun-Min Song, Wei-Tang Yuan, Hai-Jiang Wang

**Affiliations:** ^1^ Department of Anorectal Surgery, First Affiliated Hospital, Zhengzhou University, Zhengzhou 450052, China; ^2^ Department of Gastrointestinal Surgery, Affiliated Tumor Hospital, Xinjiang Medical University, Urumqi 830011, China; ^3^ Department of Orthopedic Surgery, First Affiliated Hospital, Zhengzhou University, Zhengzhou 450052, China

**Keywords:** miR-590-3p, colorectal cancer, Hippo pathway, proliferation, metastasis

## Abstract

Studies reported that miR-590-3p was involved in human cancer progression. However, its roles of oncogene or anti-oncogene in malignancies still remain elusive. This study was aimed to investigate the effect of miR-590-3p on the cell proliferation and metastasis via Hippo pathway in colorectal cancer (CRC). In our study, miR-590-3p was demonstrated highly expressed in CRC tissues, compared with adjacent normal tissues (*P*<0.05). In addition, miR-590-3p was positively associated with TNM stage and distant metastasis. Survival analysis showed that high miR-590-3p was related with poor overall survival rate. Then, over-expressed miR-590-3p was demonstrated to promote proliferation, invasion and migration of colon caner cells. What’s more, MST1, LATS1 and SAV1 mRNA were showed lowly expressed and YAP1 expression in mRNA and protein levels were highly expressed in CRC tissues, compared with adjacent normal tissues (all *P*<0.05). miR-590-3p expression was negatively associated with LATS1 and SAV1 mRNA respectively and positively related with YAP1 mRNA in CRC tissues, meanwhile, there was no relationship between miR-590-3p and MST1 mRNA. Furthermore, over-expressing miR-590-3p inhibited expressions of LATS1 and SAV1, promoted YAP1 expression and didn’t effect MST1 expression in colon cancer cells. And luciferase assay showed that miR-590-3p over-expression inhibited the luciferase activity of LATS1 and SAV1 3’UTR, meanwhile it had no effect on the mutated form of these two plasmids. Taken together, these data suggest that highly-expressed miR-590-3p promotes biological effect of proliferation and metastasis via targeting Hippo pathway, and predicts worse clinical outcomes of CRC patients.

## INTRODUCTION

Colorectal cancer (CRC) is the third most common malignant disease and remains a significant cause of morbidity and mortality worldwide [1, 2]. The global burden of CRC is expected to increase by 60% to more than 2.2 million new cases and 1.1 million deaths by the year 2030 [3, 4]. Due to its recurrence and metastasis, CRC is a leading type of cancer death, killing 26,804 males and 24,979 females in 2011 alone [5, 6].

A growing number of studies reported that microRNAs (miRNAs) are dysregulated and play a crucial role in the regulation of various biological functions of cancers [7]. Some figured out that YAP, an effector of Hippo cascade, can reprogram cancer cells into cancer stem cells and incite tumor initiation, progression and metastasis. And MST1/2, SAV1 and LATS1, in the upstream of YAP, can overexpress to down-regulate the value of YAP, which are also provided as targets for some signaling molecules [8, 9].

MiRNAs can suppress expression of target messenger RNAs (mRNAs) in post-transcriptional gene regulatory pathways and initiate either translational repression or cleavage [10–12]. Besides, alterations in miRNAs expression have been described in virtually all human cancer types [12, 13]. Accumulating researches proposed that miRNA-590-3p functioned as oncogenes or anti-oncogenes in various human cancer types. Ge et al found that over-expressed miR-590-3p enhanced the antitumor activity in the human tumor of hepatocellular carcinoma [14], and then upregulated cell migration, invasion and proliferation. However, Chen et al said that miR-590-3p might play the anti-oncogene role in prostate cancer and further repress the tumorgenesis and mobility [15]. Herein, whether miR-590-3p is a contributing factor of CRC evolution and progression or not remains controversial. The exploration of possibly regulatory mechanism still has a long way to go. Additionally, bioinformatic prediction pointed out that miR-590-3p had the potential targets of core molecules in Hippo signaling pathway, meanwhile, there are few studies on miRNAs targeting Hippo pathway to regulate cancerous clinicopathological characters by now.

With the background and hypothesis above, in our study, statistical analysis was performed to investigate expression features of miR-590-3p and core molecules of Hippo pathway, and relationship of them in CRC. And prognostic value of miR-590-3p was analyzed in CRC. Furthermore, biological function and mechanism of miR-590-3p were explored in colon cancer cells *in vitro* to verify a tumor biomarker and therapeutic target for CRC.

## RESULTS

### MiR-590-3p is highly expressed in CRC tissues and associated with poor outcome

**Figure 1:**
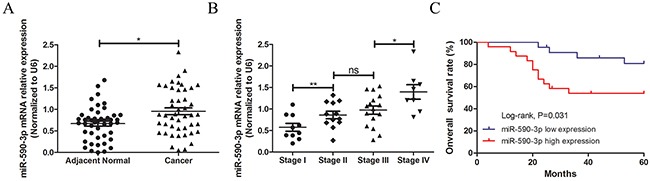
Statistical comparisons of the miR-590-3p expressions in tumor tissues and normal colorectal mucosae miR-590-3p expression level was detected usingqRT-PCR assay in a total of 46 patients with CRC (normalized to GAPDH). **(A)** miR-590-3p was highly expressed in CRC tissues, compared with adjacent normal ones. **(B)** miR-590-3p expression was displayed difference in different TNM stages of 46 CRC tissues. **(C)** Survival analysis of Log-rank assay and Kaplan-Meier curve were carried out and showed that higher miR-590-3p expression was associated with lower 5-year survival rate of patients with colorectal cancer. All graphs summarize the data from three independent experiments. ^ns^ referred to no significance, *P<0.05, ^*^P<0.01, compared with control, using Student's t-test.

In a total of 46 cases with CRC, qRT-PCR assay was carried out to detect miR-590-3p expression in cancerous tissues and their matched normal tissues, showing that miR-590-3p expression was higher in CRC tissues than that in matched normal tissues (*P*<0.05, Figure 1A).

Furthermore, to explore the expression of miR-590-3p in CRC tissues, qRT-PCR was performed in 46 pairs samples of CRC patients in diffierent TNM stages, which demonstrated that miR-590-3p expression level gradually increased along with the progression of TNM stages (all *P*<0.05, Figure 1B).

Moreover, the relationship of miR-590-3p expression and clinicopathologic features was analyzed using correlation analysis, which showed that miR-590-3p expression was positively associated with TNM stage and distant metastasis (both *P*<0.05), meanwhile, it was not related with age, gender, tumor size, tumor location and lymphatic metastasis (all *P*>0.05, Table 1).

**Table 1:** Correlation between miR-590-3p expression and clinicopathologic characteristics in CRC

	N	miR-590-3p expression		X^2^	*P* value
		Low	High		
**Age**				2.102	0.147
≤60	20	12	8		
>60	26	10	16		
**Gender**				0.382	0.536
Male	25	13	12		
Female	21	9	12		
**Tumor size (cm)**				0.425	0.515
≤5	19	8	11		
>5	27	14	13		
**Tumor location**				0.067	0.796
Rectum	20	10	10		
Colon	26	12	14		
**TNM stage**				4.224	0.040*
Stage I + II	22	14	8		
Stage III + IV	24	8	16		
**Lymphatic metastasis**				0.382	0.536
No	25	13	12		
Yes	21	9	12		
**Distant metastasis**				4.738	0.049*
No	38	21	17		
Yes	8	1	7		

Note: CRC: colorectal cancer; TNM: tumor-node-metastasis; miR-590-3p expression higher than the mean expression level was defined as high expression and otherwise low; *statistically significant.

Herein, the 46 follow-up participants with CRC were divided into two groups according to different condition of miR-590-3p expression. Then survival analysis were employed to analyze whether miR-590-3p expression was relevant to their five-year overall survival (OS) rate. Results indicated that the followed participants with high miR-590-3p expression predicted shorter OS of CRC patients than that of low miR-590-3p expression (*P*<0.05, Figure 1C).

Taken together, these data imply that over-expressed miR-590-3p might relate to tumorgenesis and often lead to poor prognosis of colon cancer cells.

### Over-expressed miR-590-3p promotes proliferation of colon cancer cells

**Figure 2:**
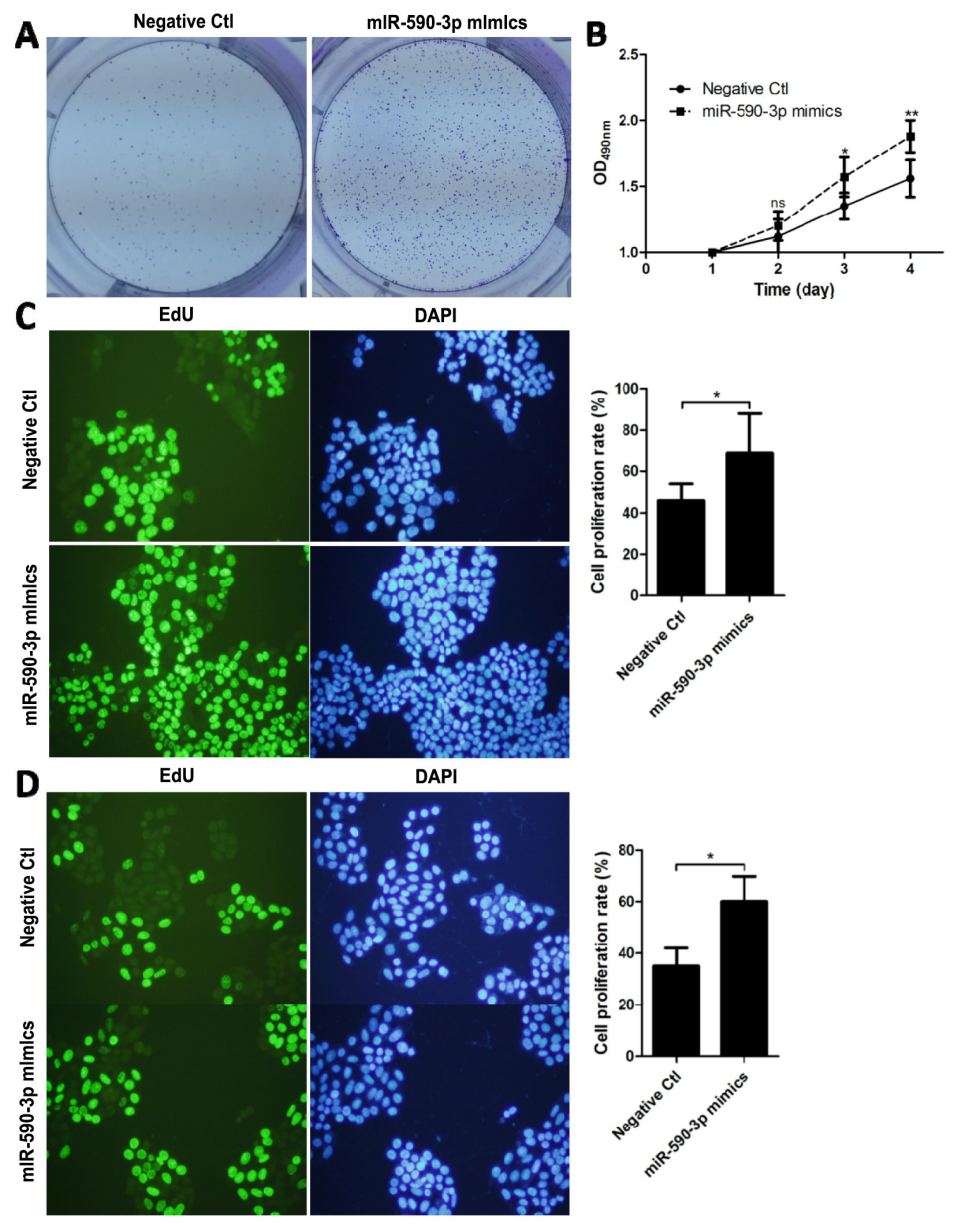
miR-590-3p mimics facilitate the proliferation of CRC cells HCT116 cells were transfected with the indicated miRNA mimics or plasmids for 24 h. **(A)** Clone formation experiments were employed between miR-590-3p mimics group and negative control group. **(B)** The transfected HCT116 cells were cultured for 4 days to perform MTT test. **(C),(D)** EdU staining and DAPI staining were detected in HCT116 and SW480 after transfection of miR-590-3p mimics or negative mimic control for 48h. Representative images (left) and quantification of migrating cells (right) are shown. All graphs summarize the data from three independent experiments. *P<0.05, ^*^P<0.01, compared with control, using Student's t-test.

In light of the above findings, we hypothesized that miR-590-3p might play a role in the tumorigenesis of CRC. HCT116 cells were transfected with miR-590-3p mimics or negative mimic control for 24h. Then, by a tumor cell clone formation assay, the distribution of HCT116 cells in miR-590-3p mimics group was more intensive than that in negative control group (Figure 2A).

Furthermore, MTT assay was performed and indicated that cell proliferation rate in miR-590-3p mimics group was higher than that in negative control group, especially after 4 days on (*P*<0.01, Figure 2B).

Then, EdU cell proliferation test was carried out and demonstrated that over-expressed miR-590-3p improved the cell proliferation rate of both HCT116 and SW480 cells. (whole *P*<0.05, Figure 2C, D).

### miR-590-3p enhances invasion and migration of colon cancer cells *in vitro*

**Figure 3:**
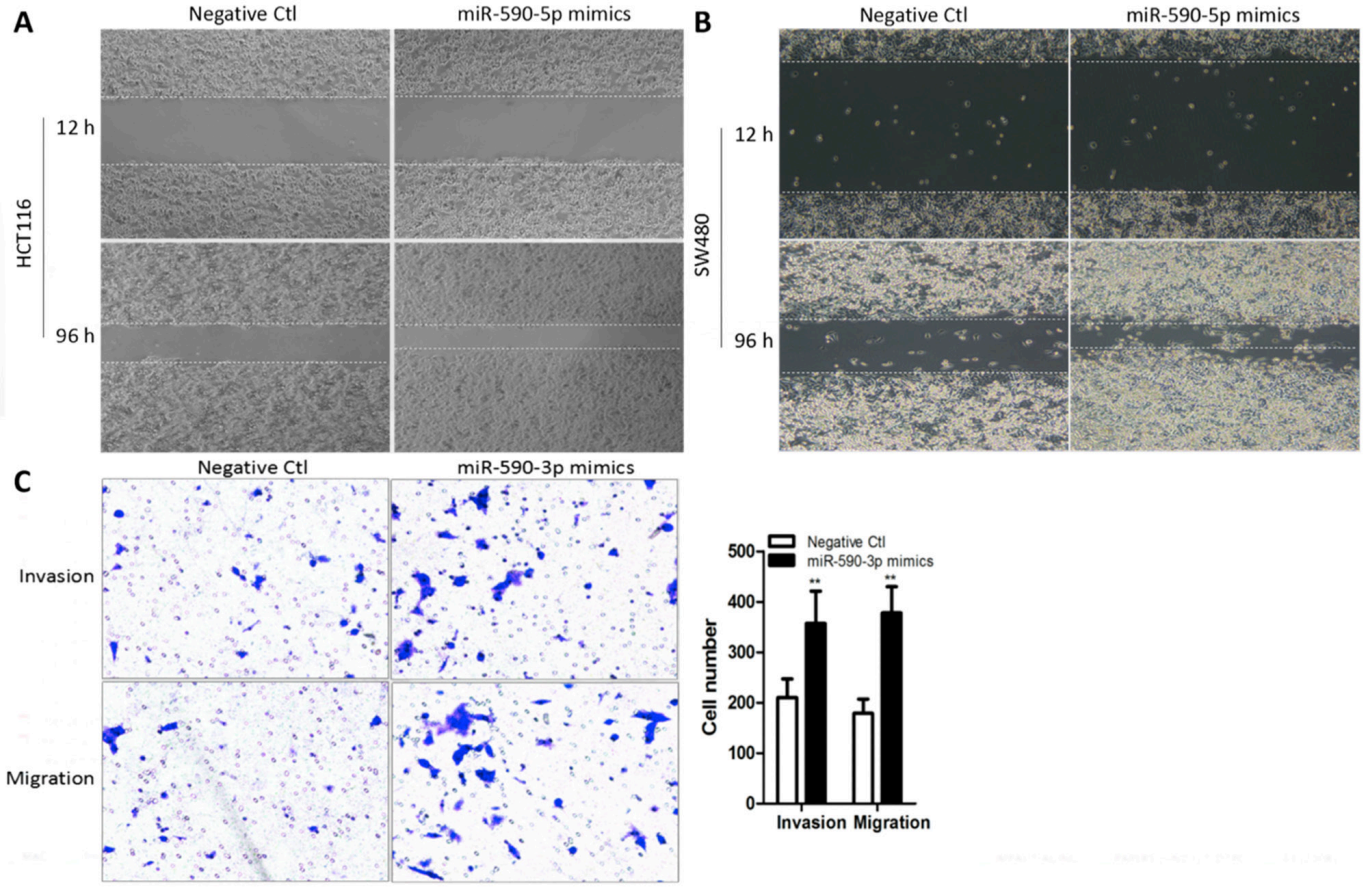
Over-expressed miR-590-3p promotes cell invasion and migration in colon cancer cells miR-590-3p mimics or negative control were transfected into HCT116 cells and SW480 cells, respectively. These transfected cells were cultured for subsequent assays. **(A),(B)** The wound healing assay was performed to evaluate migration ability of both HCT116 cells and SW480 cells. **(C)** Transwell assays were carried out to assess effect of miR-590-3p on invasion and migration capacity. The cell number of invasion and migration were presented in the right panel. All graphs summarize the data from three independent experiments. **P<0.01, compared with control, using Student's t-test.

To assess the effect of over-expressed miR-590-3p on cell invasion and migration, the wound healing assays and transwell tests were employed in transfected cells, which were same as the early transfection experiment of cell proliferation. The wound healing results showed that high miR-590-3p expression obviously enhanced the migration ability of HCT116 cells and SW480 cellss, compared with negative control (Figure 3A, B). Moreover, the results of transwell assays showed that enhancing miR-590-3 expression significantly improved the capacity of cell invasion and migration in HCT116 cells (all *P<0.05, Figure 3C).

### Expression measurement of the core factors of Hippo pathway in CRC

**Figure 4:**
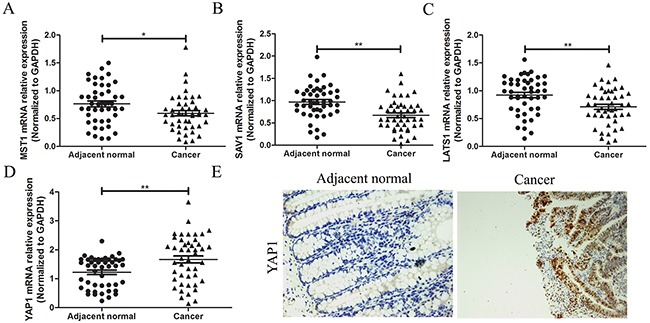
Expression measurement of the core factors of Hippo pathway in CRC A total of 46 cases of CRC tissues and adjacent normal tissues were fresh quick frozen tissues. They were used to examine expression of core factors of Hippo pathway. **(A),(B),(C)** The core factors of Hippo pathway of MST1, SAV1 and LATS1 mRNAs were measured by qRT-PCR assay and showed that they were all lowly expressed in CRC tissues, compared with adjacent normal tissues. **(D),(E) (**×200) YAP1 mRNA and protein level were detected by qRT-PCR test and immunohistochemistry assay, and indicated that YAP1 was highly expressed in CRC tissues, compared with adjacent normal tissues. All graphs summarize the data from three independent experiments. *P<0.05, ^*^P<0.01, compared with control, using Student's t-test.

Alterations of Hippo signal components connecting with tumor initiation progression was reported in various cancers. However, there was still short of evidence to exhibit expression features of Hippo pathway in CRC. Herein, we detected the expression levels of some key component expression of Hippo pathway in CRC tissues. Expression levels of MST1, SAV1 and LATS1 mRNAs were lowly expressed in CRC tissues, compared with adjacent normal tissues by qRT-PCR test (*P*<0.05 for all parameters, Figure 4A, B,C), meanwhile YAP1 mRNA and protein levels were highly expressed in CRC tissues, compared with adjacent normal tissues by qRT-PCR test and immunohistochemistry (Figure 4D, E).

### Correlation analysis between miR-590-3p expression and key molecules of Hippo pathway in CRC tissues

**Figure 5:**
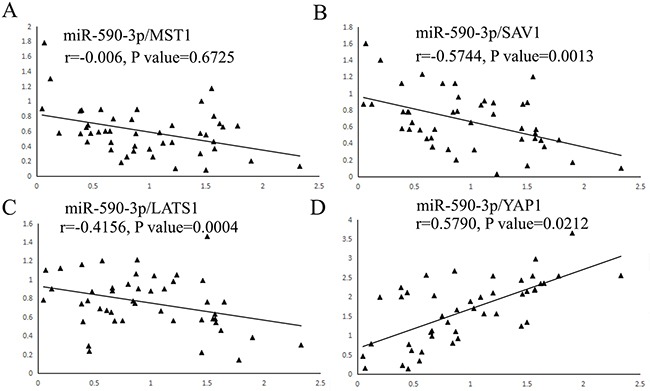
Scatter plots showing the statistical association between miR-590-3p and expression levels of key molecule mRNAs of Hippo pathway A total of 46 cases of CRC tissues and adjacent normal tissues were fresh quick frozen tissues. They were used to analyze the correlation between miR-590-3p expression and core factors of Hippo pathway by qRT-PCR assay. **(A)** miR-590-3p expression level was not related with MST1 mRNA expression in CRC tissues. **(B),(C)** The negative association between miR-590-3p and SAV1 and LATS1 mRNA expression were demonstrated respectively. **(D)** The positive association between miR-590-3p and YAP1 mRNA expression was demonstrated.

To our knowledge, there is still lack of evidence to convince if key molecules of Hippo pathway are the predicted targets of miR-590-3p. To verify the correlation between miR-590-3p and key molecules of Hippo pathway by statistical evidence, Pearson correction analysis was used. The results showed that miR-590-3p expression was not related with MST1 mRNA in CRC tissues (*P*>0.05, Figure 5A). As expected, expression levels of miR-590-3p exhibited a significant negative correlation with SAV1 mRNA (r=-0.5744, *P*<0.01, Figure 5B) and LATS1 mRNA (r=-0.4156, *P*<0.001, Figure 5C). In addition, miR-590-3p expression was positively associated YAP1 mRNA expression (r=0.5790, *P*<0.05, Figure 5D). Overall, these data suggest that SAV1, LATS1 and YAP1 have potential to be regulators of miR-590-3p in CRC.

### MiR-590-3p inhibits Hippo pathway by targeting SAV1 and LATS1 in CRC

**Figure 6:**
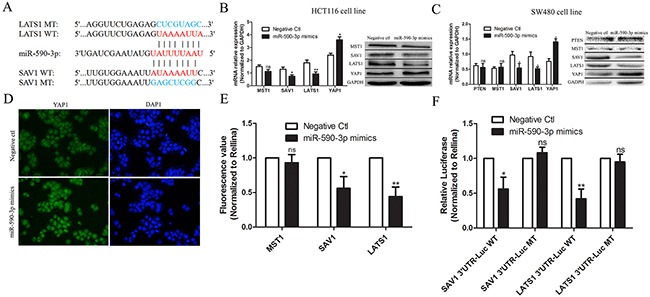
miR-590-3p inhibits Hippo pathway by targeting SAV1 and LATS1 in CRC **(A)** Bioinformatics analysis was performed and showed that 3’UTR of SAV1 and LATS1 mRNA had binding sequence of miR-590-3p. **(B),(C)** HCT116 cells or SW480 cells were transfected with miR-590-3p mimics or negative control and cultured for 48 hours. QRT-PCR and western blotting assay were carried out and the results indicated that over-expressed miR-590-3p inhibited expressions of LATS1 and SAV1, and promoted YAP1 expression (Normalized to GAPDH) in HCT116 cells or SW480 cells, respectively. **(D)** Immunofluorescent test was employed and the representative image demonstrated that over-expressed miR-590-3p improved the YAP1 expression in cell nuclear. **(E),(F)** Luciferase assay showed that miR-590-3p mimics significantly inhibited the luciferase activity of SAV1 or LATS1, meanwhile, it had less effect on the mutated form of SAV1 or LATS1 (Normalized to Rellina). All graphs summarize the data from three independent experiments. ^ns^ referred to no significance, *P<0.05, ^*^P<0.01, compared with control, using Student's t-test.

As the upstream molecules of YAP1 in Hippo pathway, low expression of MST1, SAV1 and LATS1 can reduce the level of phosphorylation of YAP1 and further enhance tissue overgrowth [9]. To demonstrate the effect of miR-590-3p on Hippo pathway, bioinformatics analysis was performed and showed that 3’UTR of SAV1 and LATS1 mRNA had binding sequence of miR-590-3p (Figure 6A). Then, qRT-PCR test and western blotting assay were performed, the results indicated that over-expressing miR-590-3p inhibited expressions of LATS1 and SAV1, promoted YAP1 expression and yet didn't effect MST1 expression in HCT116 cells and SW480 cells respectively, compared with negative control group (Figure 6B, C). Over-expressing miR-590-3p improved the expression and nuclear entry of YAP1 by cell immunofluorescence assay (Figure 6D).

Herein, to confirm the possible mechanism that miR-590-3p inhibits Hippo pathway by targeting SAV1 and LATS1, luciferase assay was preformed and showed that miR-590-3p mimics significantly inhibited the luciferase activity of SAV1 or LATS1 (Figure 6E). Furthermore, we validated the direct binding between 3’UTR of SAV1 or LATS1 mRNA and miR-590-3p at endogenous levels, and then produced luciferase constructs containing 3’UTR of SAV1 or LATS1 mRNA (SAV1 3’UTR-Luc WT or LATS1 3’UTR-Luc WT), and mutated forms (SAV1 3’UTR-Luc MT or LATS1 3’UTR-Luc MT) (Figure 6F). The data above suggests that up-regulated miR-590-3p inhibits effect of Hippo pathway via targeting SAV1 and LATS1 and promotes YAP1 expression in cell nucleus of CRC.

## DISCUSSION

MicroRNAs (miRNAs) represent a class of snRNAs that have central roles in gene silencing and function as part of large gene regulatory networks [16]. In animals, miRNAs hybridize to partially complementary binding sites that are typically located in the 3-UTR of target mRNAs and repress their expression [17]. MiRNAs are identified to have significant roles in tumorigenesis and progress, which has become a hotspot for biomedical research [14].

Studies demonstrated that miR-590-3p was dysregulated in development of some cancers and suppressed evolution and progression of hepatocellular carcinoma [14], bladder cancer [18] and glioblastoma [19], meanwhile, might play an oncogene role in prostate cancer [15]. Consequently, the detailed role of miR-590-3p in malignancies still remains controversial.

In our study, we collected 46 cases with CRC and found that miR-590-3p was over-expressed in CRC tissues. Highly-expressed miR-590-3p was associated with TNM stage and distant metastasis of CRC, which suggests that miR-590-3p might be a risk factor in tumor evolution and poor prognosis.

Mutations in Hippo pathway, firstly discovered in Drosophila melanogaster, have consistently demonstrated that dysfunctional Hippo pathway signaling led to dramatic tissue overgrowth. Rencent studies showed that all mechanisms of deregulation caused by upstream arrays resulted in the common activation of YAP [20]. Further researches proved that low expression of MST1, SAV1 and LATS1, as the upstream molecules of YAP1, can reduce the level of phosphorylation of YAP1 and enhance tissue overgrowth [9]. Through correlation analysis, we evaluated the mRNA expressed value of these key transducers in CRC tissues in comparison with adjacent normal tissues. The result implied that MST1, SAV1 and LATS1 might play an inhibitor role, whereas YAP1 acted a promoter role in the CRC evolution and progression via Hippo pathway.

In terms of mechanism, overexpression of miRNA-590-3p might promote the growth of tumor cells by targeting TFAM in colon cancer [21]. Chen et al pointed out that miR-590-3p up-regulation promoted cell proliferation and invasion by targeting inositol polyphosphate 4-phosphatase type II in human prostate cancer cells [15]. Pang et al demonstrated that miR-590-3p suppressed cancer cell migration, invasion and epithelial-mesenchymal transition in glioblastoma multiforme by inhibiting ZEB1 and ZEB2 [18]. Ge et al reported that miR-590-3p suppressed hepatocellular carcinoma growth by repressing TEAD1 [14]. Consequently, it is necessary to further explore the effect mechanism of miR-590-3p in cancers, especially for possible interacting with Hippo pathway.

Over-expression of miR-590-3p might promote tumor cell proliferation and mobility, by utilizing MTT assays and transwell assays. Moreover, Scatter plots and correlation analysis were employed that miR-590-3p had the negative association with SAV1 and LATS1, and positive association with YAP1. Interestingly, bioinformatics predicted that 3’ UTR of LATS1 and SAV1 had miRNA response element (MRE) of miR-590-3p. By immunofluorescent assay, overexpressing miR-590-3p was proved to improve the YAP1 expression in cell nuclear. Following luciferase assay demonstrated that miR-590-3p over-expression promoted the luciferase activity of LATS1 and SAV1 3’UTR, meanwhile, no effect existed in the mutated form of these two plasmids.

**Figure 7:**
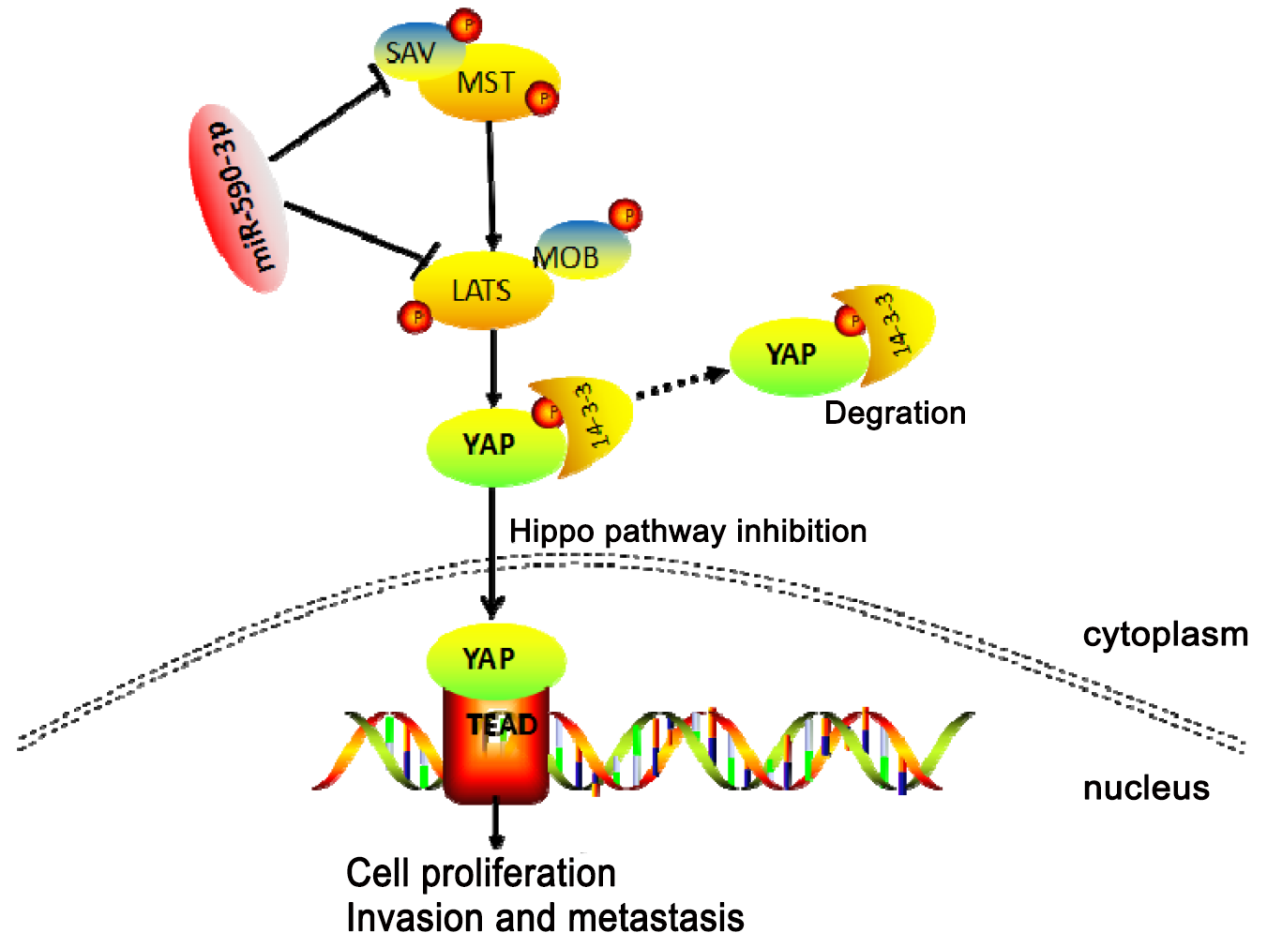
Schematic representation of a model for the major molecular mechanism of biological effect of miR-590-3p via Hippo pathway in CRC

In conclusion, these data suggested that highly-expressed miR-590-3p promoted biological effect of proliferation and metastasis via targeting Hippo pathway (Figure 7), and predicted poor clinical outcomes of CRC patients. MiR-590-3p might be a novel target for knockdown in clinical treatment. Prospectively, miR-590-3p has potential to represent a biomarker and therapeutic target for CRC in the future.

## MATERIALS AND METHODS

### Clinical research objects

A total of 46 CRC tumor samples and their matched normal tissues was collected during surgery in the First Affiliated Hospital of Zhengzhou University between 2010 Jan to 2010 Sep were enrolled. All these cases were pathologically diagnosed as adenocarcinoma. Then, these specimens were quick frozen in liquid nitrogen and further stored at −80°C for subsequent the detection of miR-590-3p expression and prognostic analysis. miR-590-3p expression was correlatively analyzed with a series of clinicopathologic data, including age, gender, tumor size, tumor location, TNM stage, lymphatic invasion and migration, distant invasion and migration and vessel invasion. The CRC were staged by the AJCC Cancer stage Manual in 2010 [22] and there were 10 cases classified as stage I, 12 cases as stage II, 16 cases as stage III, 8 cases as stage IV. None of the enrolled patients received preoperative chemotherapy, radiotherapy or immunotherapy. All tissues have been approved by the Ethic Review Committees of the First Affiliated Hospital, Zhengzhou University before usage for research. All the patients offering clinical material were signed the informed consents.

### Cell culture

HCT116 and SW480 cell lines were purchased from the cell repository of Chinese Academy of Sciences (Shanghai, China). HCT116 cell line was cultured in DMEM medium and SW480 cell line in RPMI 1640 (Gibco, Grand Island, NY, USA) medium, both with 10% fetal bovine serum (HyClone, Logan, USA) in humidified chamber at 37°C with 5% CO_2_.

### RNA isolation and qRT-PCR analysis

Total RNA was extracted using TRIzol Reagent (Invitrogen, Carlsbad, CA) and was performed the process of inverse transcription by the PrimeScript RT reagent Kit (Promega, Madison, WI, USA). The cDNA of inverse transcription product was amplified by SYBR Premix EX Taq™ (Takala, Dalian, China). Primers were as followings: miR-590-3p forward, 5’-AAAGATTCCAAGAAGCTAAGGGTG-3’and rev- erse, 5’-CCTAACTGGTTTCCTGTGCCTA-3’; MST1 forward, 5’-TAATAAGCTTATGGAACAGAAACTCATCTCTGAAG-3’ and reverse, 5’-CGATGGATCCTCAATCAGTCATGGTGCTGGCTACTC-3’; SAV1 forward, 5’-TTGGCCCTCGCCACCTAC-3’ and reverse, 5’-CCCTCCATYTCAAACACTA-3’; LAST1 forward, 5’-ACGAGGGAAAACAATAAGGG-3’ and reverse, 5’-GACAGCAAAAATCCCCTGAG-3’; YAP1 forward, 5’-CAATAGCTCAGATCCTTTCCT-3’ and reverse, 5’-TAGTATCACCTGTATCCATCTC-3’; PTEN forward, 5’-CGAACTGGTGTAATGATATGT-3’ and reverse, 5’-CATGAACTTGTCTTCCCGT-3’, GAPDH forward, 5’-TTCCGTGCTGCTCAGAAA-3’ and reverse, 5’-TGTGTTTACGAGCAGTTT-3’.

### Cell transfection

miRNA mimics and negative control were obtained from Ambion (Invitrogen, Carlsbad, CA, USA). The miRNA mimic were as follows: miRNA mimics or negative control (Rio, Guangzhou, China) was transfected into HCT116 cells or SW480 cells by Lipofectamine RNAiMAX (Invitrogen, Carlsbad, CA, USA) and Opti-MEM (Gibco, Carlsbad, CA, USA) following the manufacturer's instructions.

### Cell proliferation assay

After transfecting miR-590-3p mimics and negative control for 48h, 3,000 cells per well were grew in 96-well plates for 6 h (n=5). Then the proliferation of HCT116 cells or SW480 cells were detected by MTT assay (Roche, Basel, Switzerland) and EdU cell proliferation (Ribo, Guangzhou, China).

The transfected cells were continuously cultured for 1d, 2d, 3d, 4d and 5d, further added 10 μL MTT for 4 h, 37°C. Then removed the medium, the well were added DMSO for 10 min. The OD values were determined at 490nm by a microplate reader. In addition, the ClickREdU solution (Invitrogen, Carlsbad, CA, USA) was added to the culture medium at a ratio of 1000: 1 and the cells, which were in proliferating phase, were marked with EdU for 2 h. Similarly, the transfected cells were washed three times with PBS 0.5 g/mL DAPI (Invitrogen, Ontario, Canada) nuclei counterstained the washed cells for 10 min at room temperature in a dark. Then the DAPI-marked cells were washed three times with PBS. All marked cells were analyzed by flow cytometer FACSCalibur DXP (BD Biosciences).

### Clone formation experiment

The clone formation rate was measured by a plate clone formation assay. In total, ~200 cells were added to each well of a 6-well plate. The plates were incubated at 37°C for 14 days and gently washed and stained with crystal violet. Viable clones that contained at least 50 cells were counted.

### *In vitro* cell migration and invasion assays

The migratory ability of transfected cells was carried out using wound healing assay in a scrape, which was created by a 200 uL pipette tube. Then, the swept cells were lightly washed by PBS and serum-free medium was added to the 6-well plates. After 12 h and 96 h, the spread of wound was observed and photographed to directly assess the level of migration, respectively.

According to the experimental objective of Invasion or migration, Transwell chamber (Sigma-Aldrich Co. LLC., St. Louis, USA) was prepared with or without matrigel. Then, blood serum medium (10% FBS) was added to under chamber. Through being transfected miR-590-3p mimics and negative control, HCT116 cells were digested for preparation of cell suspension, which was added to upper chamber for 24 h Incubation. When time is up, the residual cell in upper chamber was gently wiped by cotton bud. The cells were immobilized with 4 % paraformaldehyde and stained with 1 % crystal violet for 30 min. After washed three times with PBS, the cells were imaged and counted by IX71 inverted microscope (Olympus, Tokyo, Japan). Every operation were performed three time for statistical analysis.

### Western blot analysis

The transfected cells were lysed by RIPA (Solarbio, Shanghai, China) for total protein extracts, which were quantified by BCA method. Then, the extracts were separated on 10% SDS-PAGE gel. According to sandwich structure, the electrotransfer was assembled to move the protein into PVDF membranes (GE Healthcare, Piscataway, NJ, USA), which were further incubated with the primary antibodies as follow: anti-MST1, anti-SAV1, anti-LATS1, anti-YAP1 and anti-GAPDH antibody (Cell Signaling Technology). The membranes were incubated with second antibody and measured by ECL kit (ECL Amersham).

### Immunohistochemistry (IHC)

Fixed tissue samples were sectioned at 5 μm thickness, deparaffinized and rehydrated. Then slides were placed in a solution containing 0.3% hydrogen peroxide at room temperature for 10 min and subjected to heat-induced antigen retrieval in EDTA buffer (PH 8.0) for 8 min in a microwave oven. Nonspecific staining was prevented with 10% goat serum incubation for 30 min. Afterwards, the slides were incubated at 4°C overnight with YAP (D8H1X) XP® Rabbit mAb (dilution 1:100) (#14074, Cell signaling, Danvers, MA, USA) and then with secondary antibody avidin-biotin-peroxudase-conjugated IgG at room temperature for 30 min. Proteins were visualized according to the manufacturer's instruction using 3,3’-diaminobenzidine as the substrate. The IHC results were assessed by two independent pathological investigators blinded to the patients’ and clinical status. Two independent qualified experts evaluated YAP1 expression simultaneously.

### Bioinformatics and miRNA target identification

Target prediction and functional analysis of miRNA using TargetScan, miRecods and miRDB, and the targets were validated by luciferase assay system. The luciferase assay was conducted as described by Ou [23]. In brief, a partial 3’UTR carrying predicted binding site of bovine IGFBP5 (609bp) was amplified and inserted into the 3’UTR-luciferase reporter vector (psiCHECK2 vector, Promega, Madison, WI).

### Luciferase assay

To generate the reporter vectors bearing miRNA-binding sites, wild and mutated 3’-UTR of SAV1 and 3’-UTR of LATS1 were subcloned using PCR-based methods. The constructs were inserted into the multiple cloning sites downstream of the luciferase gene in the psiCHECK-2 luciferase miRNA expression reporter vector. For the luciferase assay, 100 SAV1 3’UTR-Luc wild cells and 100 SAV1 3’UTR-Luc mutated cells were cultured to 70-80% confluence in 24-well plates, and cotransfected with miR-590-3p mimics or negative mimic control using Lipofectamine 2000 (Invitrogen), according to the manufacturer's instructions. Then, LATS1 3’UTR-Luc wild tissues and LATS1 3’UTR-Luc mutated tissues were operated in the same way. The cells were incubated with transfection reagent/DNA complex for 5 h and refreshed with fresh medium containing 10% FBS. At 48 h post-transfection, firefly and renilla luciferase activities were evaluated using the dual-luciferase reporter assay system (Promega) and the renilla luciferase activity was normalized to firefly luciferase activity.

### Immunofluorescence and image analysis

Immunofluorescence studies used YAP1 primary antibodies from Cell Signaling Technology. Coverslips were mounted with DAPI (Santa Cruz Biotechnology), and image captured with high-throughput confocal microscopy (FluoView FV1000, Olympus).

### Statistical analysis

Comparisons between groups were performed using students *t* test. The Pearson coefficient was used to assess correlalions between variables. Survival data were plotted with Kaplan-Meier curves and significance was calculated using the log-rank test. The correlations between miR-590-3p expression and clinic pathologic variables were analyzed by chi-square tests. A *P* value of <0.05 was considered to be statistically significant. All analyses were performed using the SPSS version 18.0 (SPSS, Chicago, IL, USA) or GraphPad Prism 5.

## Abbreviations

3’-UTR: 3’-untranslated region;
ANT: adjacent normal tissue;
CRC: colorectal cancer;
GEO: Gene Expression Omnibus;
IHC: immunohistochemistry technology;
miRNA: microRNA/micRNA;
LATS1: large tumor suppressor kinase 1;
miR-590-3p: Hsa-microRNA-590-3p;
MRE: miRNA response element;
NC: negative control;
OS: overall survival;
PANT: paired adjacent normal tissue;
qRT-PCR: quantitative reverse transcription polymerase chain reaction;
SAV1: salvador family domain containing protein 1;
SD: standard deviation;
TCGA: The Cancer Genome Atlas;
TNM: tumor-node-metastasis;
YAP: Yes-associated protein.
